# Assessment of a developed pig cadaver model for teaching crown lengthening surgical procedures

**DOI:** 10.7717/peerj.13421

**Published:** 2022-06-01

**Authors:** Jinsheng Zhong, Dong Shi, Cui Wang, Min Zhen, Yiping Wei, Ziyao Han, Wenjie Hu, Kwok-Hung Chung

**Affiliations:** 1 Department of Periodontology, Peking University School and Hospital of Stomatology, National Clinical Research Center for Oral Disease, National Engineering Laboratory for Digital and Material Technology of Stomatology, Beijing Key Laboratory of Digital Stomatology, Beijing, China; 2 NHC Research Center of Engineering and Technology for Computerized Dentistry, Beijing, Beijing, China; 3 Department of Restorative Dentistry, University of Washington, Seattle, United States of America

**Keywords:** Crown lengthening surgery, Animal model, Dental education

## Abstract

**Background:**

Information regarding using a pig cadaver model for teaching purposes in dentistry is limited, especially for periodontal surgery procedures. The aim of this study was to assess the feasibility and efficacy of teaching crown lengthening surgical procedures using a prepared pig cadaver model.

**Methods:**

Mandibles of slaughtered pigs with subgingival crown fracture defects on two premolars and two molars on each side were prepared as periodontal surgery teaching cases. A resident group (*n* = 20) and an instructor group (*n* = 18) participated in assessing the efficacy of the model by completing questionnaires before and after training sessions. Data was either assessed descriptively or analyzed statistically with Wilcoxon signed-rank test with the significance level at *α* = 0.05.

**Results:**

Results revealed that all the knowledge points showed statistically significant improvements (*p* < 0.05) except for the procedure to determine the quantity of bone removal during osteotomy procedures. Most residents rated the efficacy of the model obtained with 9.0 out of 10 scale. The data of effectiveness of the pig cadaver model from the instructor group ranged from 7.4 ± 1.4 to 9.0 ± 1.0.

**Conclusion:**

Results of this study support feasibility in using prepared pig cadaver models to teach crown lengthening surgical procedures to postgraduates.

## Introduction

Crown lengthening surgery is one of the most common procedures in periodontal practice. A recent American Academy of Periodontology survey reported that approximately 10% of all periodontal surgical procedures are performed in order to achieve a gain in crown length (American Academy of Periodontology, 2004). The main indications of crown lengthening surgical procedures include treatment of crown or root fractures, short clinical abutments, and subgingival caries. The rationale for crown lengthening is to reestablish the biologic width in a more apical position, in order to avoid its violation that may result in bone resorption, gingival recession, and inflammation (Palomo & Kopczyk, 1978; Marzadori et al., 2018; Pilalas, Tsalikis & Tatakis, 2016; Assif, Pilo & Marshak, 1991; Palomo & Kopczyk, 1978).

Hand-on procedures of periodontal surgery are essential for training of periodontal residents or postgraduate dentists. Working on human patients or using live animals for training purposes leads to a rise in costs and complexity (Petersilka et al., 2018). With this in mind, there are two main models for training periodontal treatments: silicone imitating models and animal models (Rühling et al., 2002; König et al., 2002; Stacy, 1967; Bonnette & Hayward, 1969; Cumming & Glavind, 1972; Al-Qareer, Afsah & Müller, 2004). Although silicone models provide a similar appearance of human periodontal tissues in order to mimic characteristics of periodontal lesions, the texture is harder and tougher than the human gingival tissue, and cannot fully simulate the true environment during periodontal surgery (Heym et al., 2016; Sunaga et al., 2013). In recent years, three-dimensional printed bone models were used as training or simulation models for tumor removal and bone reconstruction, but they were not suitable for periodontal surgery training since the periodontal soft tissue could not be printed (Meglioli et al., 2020).

High-quality simulation is critical for students to achieve adequate experience prior to clinical practice. The animal cadaver model has been used for many years in periodontal surgery teaching cases, especially using pig and sheep mandibles (Cumming & Glavind, 1972; Al-Qareer, Afsah & Müller, 2004). These models are suitable for the teaching demonstration and training of various periodontal surgeries such as gingivectomy, conventional flap operation, apically repositioned flap, coronally advanced flap, and distal wedge procedure (Al-Qareer, Afsah & Müller, 2004). Among animal tissues, periodontal tissue of pig obtained from a slaughterhouse bears excellent resemblance when compared to human histologically (Sa et al., 2016). However, without periodontal defects, the teeth in natural untreated animal cadaver models lacks the indications needed to undergo crown lengthening procedures; thus, they are not suitable cases for teaching crown lengthening procedures. In this study, subgingival marginal defects of teeth in pig cadaver models were created, which can better simulate clinical situations and provide better tactile observation of periodontal soft and hard tissues in teaching crown lengthening procedures. This model was also evaluated by all the periodontal residents at their second and third year and instructors with more than 10 years of teaching and training experience. The present study hypothesized that the designed pig cadaver models could help residents significantly improve their mastery of theoretical knowledge and clinical skill in crown lengthening surgical procedures.

## Materials & Methods

The protocol of the present study was under the exemption by the Institutional Review Board of the Peking University School and Hospital of Stomatology, with the number PKUSSIRB-202055001.

### Establishment of crown lengthening teaching models by using the pig mandible

**Figure 1:**
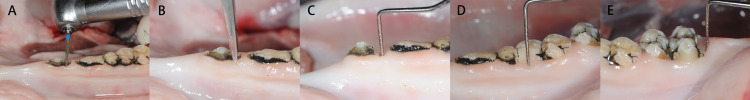
Establishment of crown lengthening models on pig cadavers. (A) A narrow deep groove was made using high-speed dental handpiece at the proximal surface of premolars. (B) The tip of dental chisel was inserted into the groove to create a tooth fracture that reaches slightly below the gingival margin. (C) Probing showed that 4 mm subgingival defect was successfully created at the mesial site of the first premolar. (D) Probing showed that 2 mm subgingival defect was successfully created at the buccal site of the first molar. (E) Probing showed that 4 mm subgingival defect was successfully created in the distal site of the most posterior molar.

**Figure 2:**
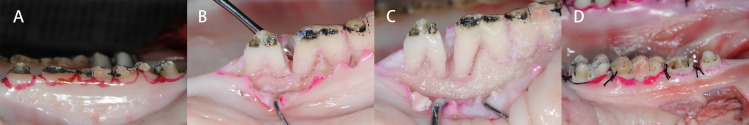
Crown lengthening surgery is demonstrated and conducted on the prepared pig mandible models. (A) During the training, the incision line was marked by red pen. (B) After finishing the incision, the flap was raised to expose the defect. (C) Ostectomy and osteoplasty were done around the teeth defects. (D) After suture, the defect was exposed.

Mandibles of slaughtered pigs were obtained and used to create crown lengthening teaching models; the preparation process is presented in Fig. 1. There are two premolars and two molars available on each side of the mandibles. Three to four defects could be made on each side: one to two defects on proximal surfaces of premolars, a defect on the buccal surface of molars, and a defect on the distal surface of the most posterior molars (Fig. 1). A narrow deep groove was made using high-speed dental handpieces (NSK, Tokyo, Japan) and diamond burs (TF-11EF, MANI, Tochigi, Japan) at the proximal and buccal surfaces of premolars and molars, as well as the distal surface of the most posterior molars. The tip of the dental chisel (#2, Kangqiao, Shanghai, China) was inserted into the groove. Light force was applied to hammer the chisel, creating a tooth fracture line reaching slightly below the gingival margin. Periodontal probing was performed by a conventional periodontal probe (Williams probes, Chicago, IL, USA) to identify the depth of the tooth defects. The depth of defects (the distance from gingival margin to the bottom of defect) varied from 2 to 4 mm, with an average of 3 mm. Using this prepared pig mandible model, crown lengthening surgery could be easily demonstrated and conducted (Fig. 2).

### Evaluation of crown lengthening procedures on the prepared pig mandible models

Both second- and third-year periodontal residents, as well as instructors with more than 10 years of teaching experience in the Department of Periodontology, Peking University School and Hospital of Stomatology, were invited to participate in this study. The residents received lectures about crown lengthening procedures before they practice on the prepared pig cadaver models. At the laboratory, instructors demonstrated the operative procedures, including the incisions, flap, osteotomy, osteoplasty, and sutures (Fig. 2). Then, residents finished the procedures by themselves. Before and after the practicing on the models, crown lengthening test was assigned to all the participants, which included both a theory section and clinical case analysis. Procedures were assessed including pre-operative analysis, incision design, osteotomy, and osteoplasty. After training with the procedures, all the participants completed their evaluation by scoring each item assessed on the teaching model (ranged from 0 (No Use) to 10 (Most Useful)) using a visual analog scale (VAS). All the instructors also finished questionnaires to assess the crown lengthening models from several aspects composing of teeth representativeness, location of the subgingival defects, match with the theory, detailed operation procedures, and the overall teaching effectiveness.

### Statistical analysis

All the data were stored using Excel 2016 (Microsoft Corporation) and analyzed using SPSS 26.0 (IBM Corporation). To compare the test results of the residents before and after training, the Shapiro–wilk test was made to test the data normality, and a paired-*t* test or Wilcoxon signed-rank test was performed based on the normality of the data. Statistical significance was represented at *p* < 0.05.

## Results

Twenty residents and eighteen instructors participated in assessing and practicing on the prepared pig model. The data of the residents scores did not conform with normality; the Wilcoxon signed-rank test was performed to compare the test results of the residents before and after training. It demonstrated that the master of incision design of the anterior, premolar, molar teeth (*p* < 0.001) and osteoplasty in posterior area (*p* < 0.001) and preoperative analysis (*p* = 0.001) and osteoplasty in anterior area (*p* = 0.002) were significantly improved after training, except for the amount of osteotomy procedures (*p* = 0.157) (Table 1). This indicated that residents knew the basic knowledge of biological width well before they did the training on the models. The results of VAS scores showed that residents gave high evaluation scores of 9.0 out of 10 in regards to the model and training session helping them master the theory and technique of crown lengthening surgery. Most residents thought the training model could help them improve their skills of crown lengthening surgery, while some residents suggested that the complex shape of porcine teeth made the surgery more difficult.

**Table 1:** Test results before and after training by crown lengthening models (Mean ± SD).

Subject	Max score	Score before training	Score before training/ Max score (%)	Score after training	Score after training/ Max score (%)	△ Score (after –before training)	*P value*
Pre-operative analysis	20	13.2 ± 2.4	66 ± 12	16.4 ± 2.2	82 ± 11	3.2 ± 2.8	0.001^*^
Incision design (anterior teeth)	15	8.6 ± 4.8	57.3 ± 32	13.5 ± 1.6	90 ± 10.7	5.0 ± 3.9	<0.001^*^
Incision design (premolar)	15	8.4 ± 3.2	56 ± 21.3	12.5 ± 1.8	83.3 ± 12	4.1 ± 2.9	<0.001^*^
Incision design (molar)	15	5.4 ± 2.0	36 ± 13.3	11.7 ± 1.8	78 ± 12	6.3 ± 2.7	<0.001^*^
Amount of osteotomy	15	14.3 ± 2.4	95.3 ± 16	15 ± 0.0	100 ± 0	0.8 ± 2.4	0.157
Osteoplasty in anterior area	10	5.5 ± 2.4	55 ± 16	7.7 ± 0.9	77 ± 9	2.2 ± 2.5	0.002^*^
Osteoplasty in posterior area	10	2.4 ± 2.0	24 ± 20	7.8 ± 0.9	78 ± 9	5.4 ± 2.3	<0.001^*^
Total	100	57.7 ± 8.9	55.6 ± 9.2	84.6 ± 5.4	84.6 ± 5.5	26.9 ± 10.0	<0.001^*^		

**Notes.**

* Significant difference at *P* < 0.05.

The questionnaire scores from the instructors ranged from 7.4 to 9.0, indicating that teaching crown lengthening surgical procedures using this model was feasible and highly recommended (Table 2). Some instructors thought that the effect of this model for training esthetic crown lengthening surgery still needed to be investigated.

**Table 2:** The questionnaires of 18 instructors about the usefulness of the crown lengthening model.

Subject	Score^*^
Teeth representativeness	8.1 ± 1.0
Location of the defect below the gingiva	7.4 ± 1.4
Match with the theory	9.0 ± 1.0
Detailed operation procedure	8.2 ± 1.9
Overall teaching effectiveness	8.8 ± 1.3

**Notes.**

* 0, no use; 10, most useful.

## Discussion

The current study showed that subgingival defects could be easily and successfully created in the mandible of a pig cadaver to stimulate the clinical situation required in crown lengthening surgical procedures. Three to four defects can be created on each side of one mandible, which can provide different operative strategies. One pig mandible could be fully utilized to serve many crown lengthening surgical procedure teaching cases.

Previous studies showed that animal models could be useful in the periodontal surgery teaching (Cumming & Glavind, 1972; Al-Qareer, Afsah & Müller, 2004). Severe periodontal diseases could be found on these natural dentitions of some old animal models (Cutress & Ludwig, 1969). Therefore, for educational purposes, teaching periodontal surgery such as flap operation, coronally advanced flap, gingivectomy, and distal wedge procedure could be conducted on dentitions of animal models without prior preparation. However, crown lengthening surgery training requires subgingival tooth defects, which were rarely found on the natural dentitions and needed to be artificially created. By creating subgingival tooth defects on pig mandible, the prepared models successfully simulated clinical situations and demonstrated success in crown lengthening training and practice. The training model achieved high evaluation scores from the residents (9.0 out of 10 scales) and the instructors (8.8 out of 10 scales), which indicated that the training model was useful in helping residents master and understand the crown lengthening surgery better. To our knowledge, this is the first cadaver model specifically designed for the training of crown lengthening surgery techniques.

According to the procedures of crown lengthening surgery, seven evaluation aspects were extracted to check the mastery of the surgery. The results showed that after the model training, six aspects (preoperative analysis, incision design (anterior teeth), incision design (premolar), incision design (molar), osteoplasty in anterior area, osteoplasty in posterior area) had been significantly improved. The residents had already understood the basic theory of biological width, and most of them kept in mind that the defect margin should be at least three mm away from the underneath alveolar bone after the lecture; this could explain why the students’ score did not improve when it came to the osteotomy procedure itself. Therefore, the null hypothesis was partially rejected. However, after training on the pig mandible model, the residents showed a better understanding of the procedures of crown lengthening surgery, which was important for a resident to transfer the theoretical knowledge into clinical practice.

The instructors also gave high evaluation on the model. It was found that the appropriate depth of the defect could be simply created by using a high-speed handpiece and chisel, controlled within 3 ± 1 mm. Therefore, high reproducibility could be achieved on the preparation of this model, which makes it feasible to promote the model for the training of crown lengthening surgery.

## Conclusions

The present study suggested that the pig mandible model can be a feasible, effective, and well-accepted simulation for the teaching and training of crown lengthening surgical procedures.

## Supplemental Information

10.7717/peerj.13421/supp-1 Supplemental Information 1 The raw data of questionnaires answered by 10 students

10.7717/peerj.13421/supp-2 Supplemental Information 2 The raw data of questionnaires answered by 18 instructors about the usefulness of the teaching model

10.7717/peerj.13421/supp-3 Supplemental Information 3 Evaluation questionnaire for teachers

10.7717/peerj.13421/supp-4 Supplemental Information 4 Evaluation questionnaire for students

10.7717/peerj.13421/supp-5 Supplemental Information 5 Evaluation questionnaires for teachers (Chinese)

10.7717/peerj.13421/supp-6 Supplemental Information 6 Evaluation questionnaires for students (Chinese)

## Additional Information and Declarations

### Data Availability

The following information was supplied regarding data availability: The raw measurements are available in the Supplementary Files.
