# Supplementary material for: Assessment of a developed pig cadaver model for teaching crown lengthening surgical procedures
Source: PeerJ. 2022 Jun 1;10:e13421. doi: 10.7717/peerj.13421 (PMC9166679; doi:10.7717/peerj.13421)
Supplement: Supplemental Information 3 [file peerj-10-13421-s003.docx]

**Evaluation questionnaire of the crown lengthening teaching model and training method (for teachers)**

1. How are the clinical representativeness of the three tooth positions chosen for the surgical model? (VAS score)

10

0

1. In this surgical model, how well does the subgingival position of the edge of the crown fracture simulate clinical circumstances? (VAS score)

0

10

1. Do the theoretical courses cover the basic theoretical knowledge of crown lengthening surgery? (VAS score)

0

10

1. Whether the surgical training methods of using animal models cover the technical points of crown lengthening surgery? (VAS score)

0

10

1. Could preclinical training of crown lengthening for postgraduates be achieved by using this surgical model?

10

0

1. Do you have any suggestions for the improvements of the teaching model？
