# Supplementary material for: Assessment of a developed pig cadaver model for teaching crown lengthening surgical procedures
Source: PeerJ. 2022 Jun 1;10:e13421. doi: 10.7717/peerj.13421 (PMC9166679; doi:10.7717/peerj.13421)
Supplement: Supplemental Information 4 [file peerj-10-13421-s004.docx]

**Evaluation questionnaire of the crown lengthening teaching model (for students)**

1. Please try to answer what factors should be considered in the design before crown lengthening?
2. In this picture, the 23 was a residual root and the edge of the fracture was at the same level or 1 mm below the gingival margin, which needed to receive crown lengthening surgery. The edge of the fracture should be exposed with 1 mm beyond the gingival margin postoperatively, please draw the labial incision line in the picture below (text description was allowed).

| 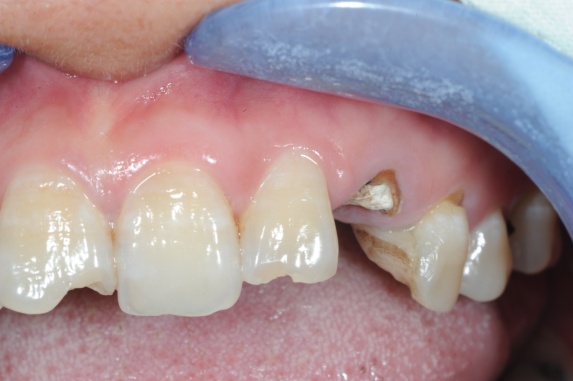 | 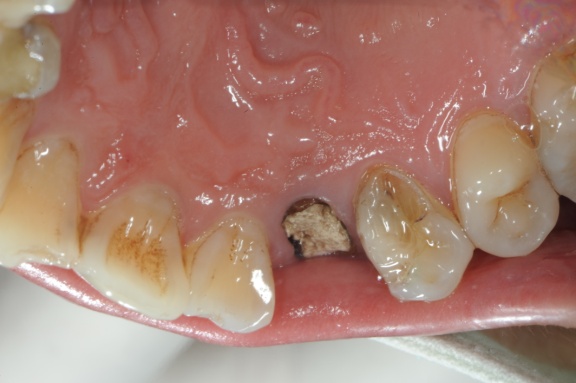 |
| --- | --- |

1. The pictures showed the crown fracture of 25, and the edges of the fracture were 2-3 mm below the gingival margin at interproximal and palatal sites, which needed to receive crown lengthening surgery. The edge of the fracture should be exposed with 1 mm beyond the gingival margin postoperatively, please draw the incision line in the picture below (text description was allowed).

| 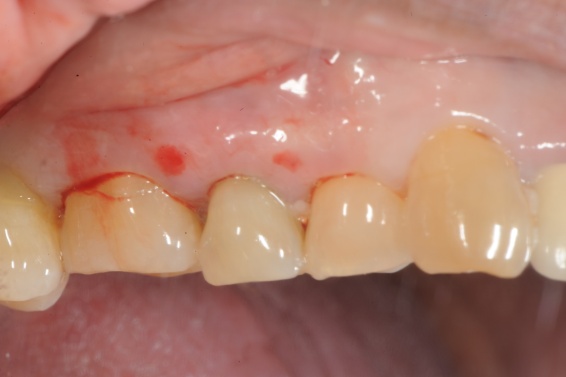 | 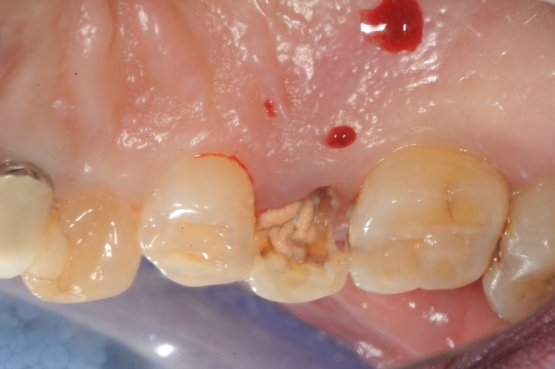 |
| --- | --- |

1. The pictures showed the crown fracture of 47. The edges of the fracture were 2 mm below the gingival margin at the distal-buccal sides and at the same level with the gingival margin at distal side. The edge of the fracture should be exposed with 1 mm beyond the gingival margin postoperatively, please draw the incision line in the picture below (text description was allowed).

| 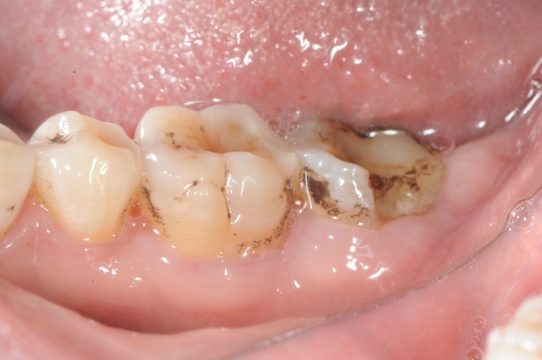 | 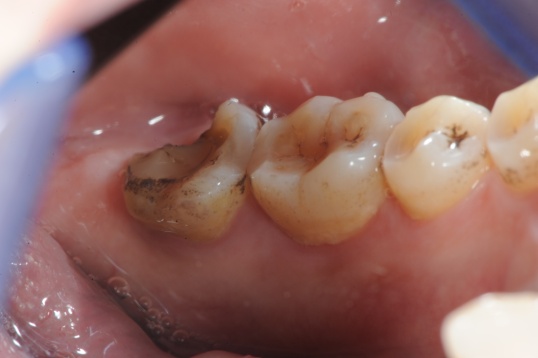 |
| --- | --- |

1. The edge of the fracture was 2 mm beyond the alveolar crest, if the edge of the fracture should be exposed with 1 mm beyond the gingival margin after crown lengthening surgery, please give the amount of osteotomy procedures and your evidence.
2. When performing the crown lengthening surgery in the esthetic area, please try to describe the ideal morphology at labial and interproximal sides after osteotomy in words or drawings.
3. When performing the crown lengthening surgery in the molar area, please try to describe the ideal morphology at labial and interproximal sides after osteotomy in words or drawings.
4. Please try to score how this crown lengthening surgical model and training mode help you master and understand the theory and operation of crown lengthening surgery? (VAS, after class)

10

0

1. Do you have any suggestions for the surgical model？
