# Supplementary material for: Assessment of a developed pig cadaver model for teaching crown lengthening surgical procedures
Source: PeerJ. 2022 Jun 1;10:e13421. doi: 10.7717/peerj.13421 (PMC9166679; doi:10.7717/peerj.13421)
Supplement: Supplemental Information 5 [file peerj-10-13421-s005.docx]

**牙冠延长手术教学动物模型及训练方式评价问卷（教师用）**

1. 该手术模型所设计的三个牙位是否具有临床代表性？（VAS评分）

10

0

1. 该手术模型中，牙冠劈裂断缘在龈下的位置模拟临床的程度如何？（VAS评分）

10

0

1. 理论课的内容是否涵盖了牙冠延长术的基本理论知识点?（VAS评分）

10

0

1. 使用动物模型的手术训练方式是否涵盖了牙冠延长手术的技术要点？（VAS评分）

10

0

1. 利用该手术模型对研究生进行牙冠延长术的临床前培训是否可以达到教学目的?（VAS评分）

10

0

1. 您对该模型还有何改进建议？
