# Supplementary material for: Assessment of a developed pig cadaver model for teaching crown lengthening surgical procedures
Source: PeerJ. 2022 Jun 1;10:e13421. doi: 10.7717/peerj.13421 (PMC9166679; doi:10.7717/peerj.13421)
Supplement: Supplemental Information 6 [file peerj-10-13421-s006.docx]

**牙冠延长手术教学模型评价问卷（学生用）**

1. 牙冠延长手术前设计时应考虑那些因素，请尝试作答。
2. 图中患者左上3残根，断端齐龈或位于龈下1mm处，拟行牙冠延长术，要求术后断缘暴露在龈上1mm，请在下图中画出唇侧切口线。（可加文字描述）

| 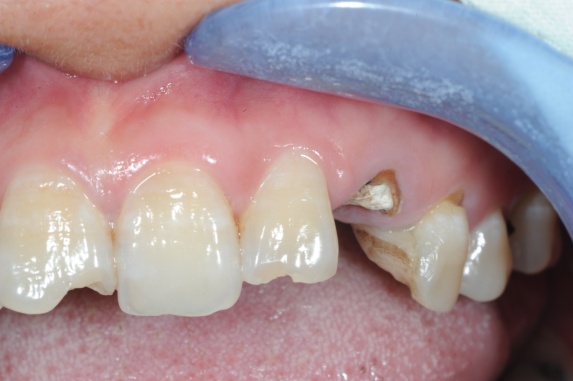 | 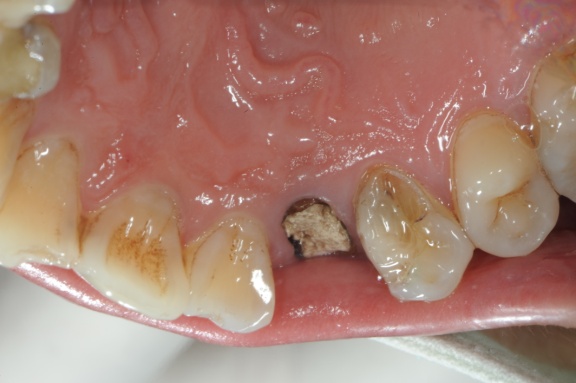 |
| --- | --- |

1. 图中患者左上5劈裂，腭侧及近远中断缘位于龈下2-3mm处，拟行牙冠延长术，要求术后断缘暴露在龈上1mm处，请在下图中画出切口线。（可加文字描述）

| 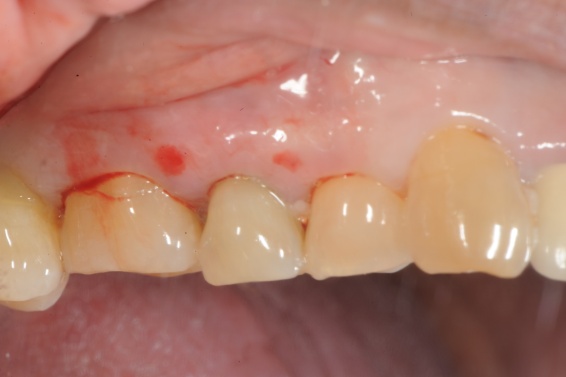 | 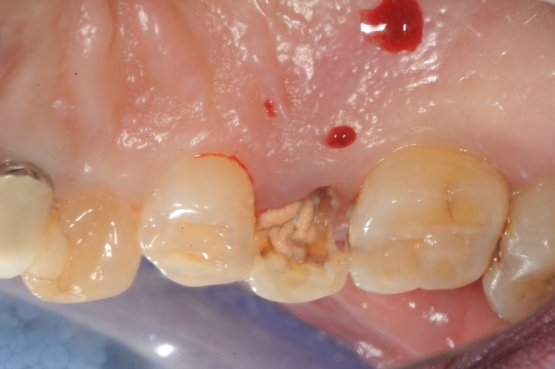 |
| --- | --- |

1. 图中患者右下7劈裂，颊侧偏远中断端位于龈下2mm处，远中咬合面齐龈，要求暴露断端1mm，远中延长临床牙冠2mm，请在图中画出切口线。（可加文字描述）

| 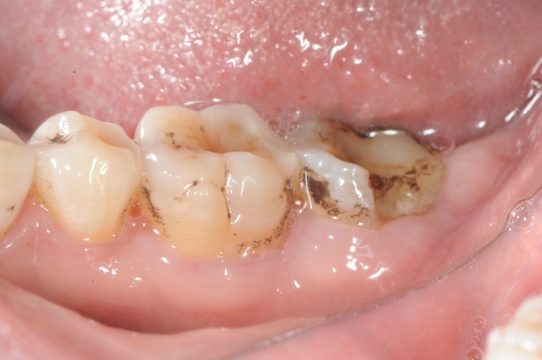 | 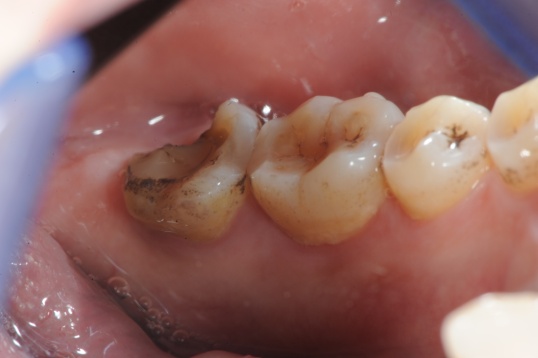 |
| --- | --- |

1. 牙冠延长术中，断缘位于牙槽嵴顶冠方2mm，如果要求术后断缘暴露在龈上1mm处，去骨量应为多少，并给出依据。
2. 前牙冠延长手术中，唇侧和邻面去骨后应该达到怎样的形态标准，请尝试用文字或绘图的形式进行描述。
3. 磨牙冠延长手术中，唇侧和邻面去骨后应该达到怎样的形态标准，请尝试用文字或绘图的形式进行描述。
4. 本牙冠延长术模型对于你掌握和理解牙冠延长术理论和操作有多大帮助，请尝试对本手术模型和训练模式进行评分（VAS评分，课后进行）。

10

0

1. 你对该手术模型还有何建议？
